# Case of Ascites

**Published:** 1803-03-01

**Authors:** 

**Affiliations:** House Surgeon to the General Infirmary, Sheffield


### Case *of* Ascites; *communicated by* Mr. Earnest, *House Surgeon to the General Infirmary, Sheffield.*

THE following case is intended, by one more example, to shew the necessity principiis obstandi. If it has that effect, I shall be sufficiently rewarded.

E. T. ætat. 27, of a spare habit and florid complexion, during the menstruating period, was exposed for ten hours to the rain. The evacuation ceased immediately and never afterwards returned. A pain took place on the left side of the abdomen; loss of appetite and spirits, succeeded with thirst; respiration frequent and incommoded; pulse frequent, weak, hard, and small; and debility in motion. Six weeks afterwards an hard tumour was felt in the pained place, and soon to this succeeded a suppression of urine and a fullness in the lower part of the abdomen, which increased so fast that in the twentieth week afterwards she was admitted into the Sheffield General Infirmary, as a dropsical patient.

The various evacuants having been given in vain, recourse was had to the paracentesis, and the annexed table will shew the number of operations, and the quantity and quality of the fluid evacuated.

The general symptoms above enumerated continued nearly stationary till about the twentieth operation, when they began to increase, and, six days before death, occasional nausea, vomiting, and syncope took place; in the end she expired suddenly.

The succeeding extispicium pointed out the material cause of the disease to be that part of the mesentery which supports the colon sinistrum or descendens, elevated into a tumour, weighing eight pounds, partly scirrhous, partly apostemous;* the consequences of which, more or less remote, were an adhesion of the convex side of the liver to the peritonæum; an adhesion to that part of the lesser intestine near the tumour to the peritonæum, and the surface of the peritonæum itself, to be covered with the coagulable part of the pus, where it touched the tumour.† All the other parts were natural.

If

* Whenever I speak of pus in this history, I mean a puriform mucus not readily miscible in water.

† Doubt arising whether the pus found on the peritonæum might not have been formed there, it was carefully washed off, and the membrane was sound.

If I mistake not, this disease must be considered as an individual of the Ascites Mesentericus.

*Jan.* 27, 1803.

### Table of Operations, &c.

| No. of Operat. | Date. | Quantity of Fluid evacuat. | Quality of the Fluid. |
|---|---|---|---|
| | 1800. | | |
| 1 | March 21, | 36 pints, | Mucilage much, Pus some. |
| 2 | May 28, | 32 pints, | ——— less, — more. |
| 3 | Aug. 4, | 40 pints, | ——— less, — more. |
| 4 | Oct. 6, | 53 pints, | ——— more, — more. |
| 5 | Dec. 1, | 60 pints, | ——— more, — less. |
| | 1801. | | |
| 6 | Jan. 29, | 70 pints, | ——— less, — more. |
| 7 | March 29, | 65 pints, | ——— more, — less. |
| 8 | May 25, | 68 pints, | ——— more, — less. |
| 9 | July 23, | 74 pints, | ——— less. — more. |
| 10 | Sept. 19, | 76 pints, | ——— less, — more. |
| 11 | Oct. 28, | 72 pints, | ——— less, — more. |
| 12 | Dec. 7, | 72 pints, | ——— more, — less. |
| | 1802. | | |
| 13 | Jan. 19, | 66 pints, | ——— more, — less. |
| 14 | Feb. 20, | 68 pints, | ——— more, — less. |
| 15 | April 3, | 69 pints, | ——— less, — more. |
| 16 | May 16, | 60 pints, | ——— more, — more. |
| 17 | June 21, | 64 pints, | ——— more, — more. |
| 18 | July 26, | 76 pints, | ——— less, — more. |
| 19 | Aug. 25, | 72 pints, | ——— less, — more. |
| 20 | Sept. 21, | 58 pints, | ——— more, — more. |
| 21 | Oct. 19, | 54 pints, | ——— more, — more. |
| 22 | Nov. 15, | 52 pints, | ——— more, — more. |
| 23 | Dec. 7, | 49 pints, | ——— much more — more. |
| Eight pints were found extravasated into the abdomen, when opened. | | | |

Pints 1414 ÷ 8 = 176 gallons 6 pints.

